# Visual-Gustatory Interaction: Orbitofrontal and Insular Cortices Mediate the Effect of High-Calorie Visual Food Cues on Taste Pleasantness

**DOI:** 10.1371/journal.pone.0032434

**Published:** 2012-03-14

**Authors:** Kathrin Ohla, Ulrike Toepel, Johannes le Coutre, Julie Hudry

**Affiliations:** 1 Perception Physiology, Nestlé Research Center, Vers-chez-les-Blanc, Lausanne, Switzerland; 2 Departments for Clinical Neurosciences and Radiology, Centre Hospitalier Universitaire Vaudois and University of Lausanne, Lausanne, Switzerland; 3 Organization for Interdisciplinary Research Projects, The University of Tokyo, Tokyo, Japan; German Institute for Human Nutrition, Germany

## Abstract

Vision provides a primary sensory input for food perception. It raises expectations on taste and nutritional value and drives acceptance or rejection. So far, the impact of visual food cues varying in energy content on subsequent taste integration remains unexplored. Using electrical neuroimaging, we assessed whether high- and low-calorie food cues differentially influence the brain processing and perception of a subsequent neutral electric taste. When viewing high-calorie food images, participants reported the subsequent taste to be more pleasant than when low-calorie food images preceded the identical taste. Moreover, the taste-evoked neural activity was stronger in the bilateral insula and the adjacent frontal operculum (FOP) within 100 ms after taste onset when preceded by high- *versus* low-calorie cues. A similar pattern evolved in the anterior cingulate (ACC) and medial orbitofrontal cortex (OFC) around 180 ms, as well as, in the right insula, around 360 ms. The activation differences in the OFC correlated positively with changes in taste pleasantness, a finding that is an accord with the role of the OFC in the hedonic evaluation of taste. Later activation differences in the right insula likely indicate revaluation of interoceptive taste awareness. Our findings reveal previously unknown mechanisms of cross-modal, visual-gustatory, sensory interactions underlying food evaluation.

## Introduction

One of the properties of food accounting for its palatability is energy density, *i.e.* calorie content. The energetic content of visually presented food is evaluated in an automatic and fast manner [1], [2] corroborating the greater motivational salience of high-calorie food [3]. There is evidence that the mere viewing of food images activates taste-related brain areas and elicits expectations about the taste and hedonic aspects of the food [1], [3], [4]. This is not surprising because visual cues constitute a primary sensory input indicating the pre-ingestive availability and palatability of food.

Notwithstanding, the sense of taste provides a major input for food perception. When presented alone, taste activates a cortical network including the insula/frontal operculum (FOP), orbitofrontal cortex (OFC), and anterior cingulate cortex (ACC) [5]–[10]. The human insula is involved in the processing of various sensory food properties including taste [11], oral texture [12], and oral temperature [13], but it is also believed to integrate multisensory information to establish an emotionally relevant context for sensory experience [14]. The OFC has been particularly associated with hedonic aspects of sensory experience, the processing of food reward, and positive reinforcement irrespective of stimulus modality [9], [15]–[19].

In humans, responses of the insula are correlated with the subjective intensity of taste, while those of the OFC are correlated with the subjective pleasantness of taste [20]. Yet, when altering the value of a sweet taste stimulus using an aversive cue, taste representation has been found modified already at an early processing level, as indicated by suppressed responses to the tastant in rat primary gustatory cortex [21]. Similarly, it has been proposed that the primary gustatory cortex jointly encodes both the chemical identity and palatability of a tastant [22] suggesting a role of the insula in the evaluation of taste or its precursors beyond mere sensory processing.

The consequences of visual energy cues on taste processing remain unexplored. Therefore, using electro-encephalography (EEG), we investigated whether images of high- and low-calorie food differentially influence the processing of subsequent electric tastes. Electric taste, as generated by a small current on the tongue, elicits event-related potentials (ERPs) with the first positive peak occurring around 130 ms, followed by a central negativity at around 220 ms and a long-lasting central-parietal positivity [23]. Similar waveforms have been described in ERP studies using chemical tastants, with yet a variable timing depending upon the stimulus intensity and quality as well as the device used for its delivery [24]–[29]. Information about topography of the peaks obtained with chemical tastants is scarce as taste ERP studies only used a small number of electrodes distributed on the central midline or in the fronto-central region [30].

Electric taste is unique in that it is hedonically neutral – neither very pleasant nor unpleasant – and its quality is unrelated to any food image content. In addition, when presented at detection threshold level, electric taste activates a dynamic network encompassing key gustatory brain areas, namely the insula, the opercula, and the ventromedial OFC [23]. We hypothesize that high-calorie food cues enhance the hedonic evaluation of the consecutive taste and increase activation in reward-related cortical regions.

## Materials and Methods

### Participants

Fourteen healthy participants (9 males), aged 22–30 (mean 25.4 yrs), were recruited and paid for participation. Participants went through a medical screening and signed informed consent prior to the experiment. The study protocol was approved by the local ethics board (“Commission cantonale d'éthique de la recherche sur l'être humain”, Lausanne, Switzerland) and conformed to the revised Declaration of Helsinki.

### Stimuli and procedures

The visual stimuli were 150 photographs depicting 100 food items and 50 non-food kitchen utensils [2], [31]. The food images were subdivided into high-calorie and low-calorie classes based on the energy content obtained from the United States Department of Agriculture (www.nal.usda.gov/fnic) and the Swiss nutritional database (released by the Swiss Federal Office of Health and the Swiss Federal Institute of Technology Zurich). The energy content of the low-calorie and high-calorie group ranged from 12–151 kcal/100 g (mean = 66.18, SEM = 6.50) and 160–717 kcal/100 g (mean = 352.50, SEM = 17.32), respectively. The caloric content of the high-calorie foods was significantly greater than that of the low-calorie foods (t_2,98_ = 15.48, p<0.001).

**Figure 1:**
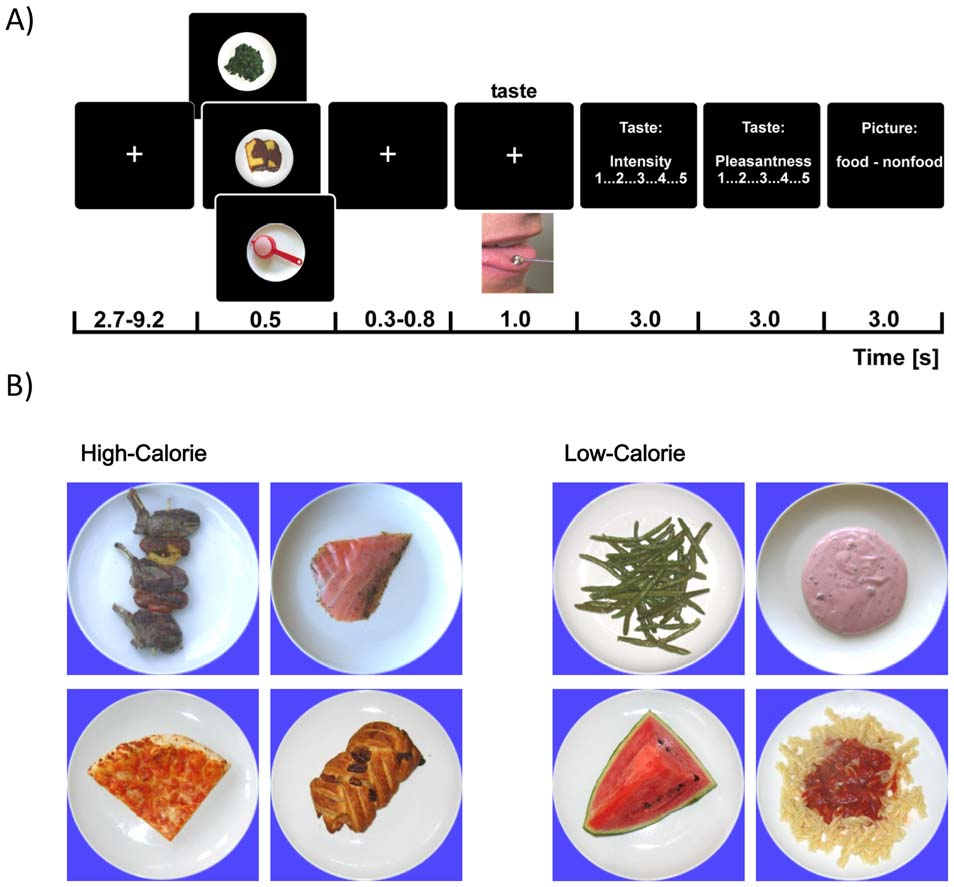
Experimental design. (A) Each trial started with the presentation of a fixation cross followed by an image of either a high-calorie or a low-calorie food or a non-food item. Following a variable inter-stimulus interval, a neutral taste stimulus was presented. After the offset of the taste, participants had to first rate the taste intensity and pleasantness and then categorize the image preceding the taste into food *vs.* non-food items. They were naïve as to the task-irrelevant, *i.e.* high- and low-calorie image categories. (B) To illustrate the variety of high-calorie stimuli (160–717 kcal/100 g) and low-calorie stimuli (12–151 kcal/100 g), four pictures of each category are presented. High-calorie: Lamb-chops, Salmon, Pizza, Pastry. Low-calorie: Beans, Water melon, Yoghurt, Pasta w. Tomato Sauce.

The taste stimuli were single square wave anodal pulses of 1000 ms duration applied via a stainless steel electrode (approx. 5 mm in diameter) connected to the RION TR-06 gustometer (Sensonics, Inc., NJ, USA). The electrode (anode) was placed on the anterior edge of the tongue surface; the cathode was placed on the left upper arm. Participants held the electrode gently between their teeth while keeping the mouth closed. The electrode remained on the tongue throughout experimental blocks to minimize thermal and tactile effects from the placement [23]. The taste intensity was set for each participant to the individual threshold and kept constant throughout the experiment. Taste detection thresholds were assessed via a computer-controlled ascending staircase procedure. For this, the intensity was increased in steps of 2 db until the first correct trial. Reversal points were determined by three correct responses at a given concentration when moving up the staircase and a single incorrect response when moving down the staircase. After five reversals or a maximum of 40 trials, the threshold was defined. Only participants whose thresholds could be unequivocally determined and who reported a clear taste sensation, using an established list of descriptors [32], were included in the EEG study. A schematic view of a trial is shown in Figure 1. Participants viewed a central white fixation cross on a black background presented on a 17″ TFT screen at a 80 cm distance. Fixation was followed by an image of either of three image categories (high-calorie foods, low-calorie foods or non-food) that was presented for 500 ms. Following a randomized inter-stimulus interval of 300–800 ms, the electric taste stimulus at individual threshold intensity was delivered for 1000 ms. Each taste stimulus was evaluated for its intensity and pleasantness on a 5-point Likert scale ranging from 1 to 5 immediately after stimulus offset. Participants were blind to the fact that all taste stimuli were identical throughout the experiment. On each trial, participants had to categorize images into food *vs.* non-food objects. Thus, participants remained naïve with respect to the implicit high- *vs.* low-calorie subcategory of food images. The taste onset asynchrony varied between 14–20 s. The experiment was separated by breaks during which participants ingested 30 ml of mineral water (Nestlé Aquarel) to minimize effects of varying oral moisturization. Furthermore, participants refrained from eating and drinking (only water was allowed) two hours before the testing and additionally rinsed their mouth before the start of the experiment.

### Data acquisition and pre-processing

The experiment was conducted in an electrically shielded and sound-attenuated recording booth. EEG was continuously recorded with a BioSemi Active-Two amplifier system (BioSemi, Amsterdam, The Netherlands) using 64 Ag/AgCl active electrodes mounted in an elastic cap and placed according to the international extended 10–20 system. During the recordings, the signals were referenced to CMS (common mode sense), while DRL (driven right leg) served as ground (for details see http://www.biosemi.com/faq/cms&drl.htm). Data were recorded with a sampling rate of 512 Hz and analog filtered (0.06 and 200 Hz). The continuous EEG signal was stored on hard disk for off-line analysis. EEG data were processed using EEGLAB [33] running under MATLAB (Mathworks, Natick, MA, USA) and Cartool (http://sites.google.com/site/fbmlab/cartool). Data were segmented into epochs from −500 to 1000 ms relative to the onset of the taste stimulus. Epochs with unique, non-stereotypic artifacts were manually rejected. Then, extended infomax ICA, as implemented in EEGLAB, was applied to the remaining concatenated single trials. Independent components representing common EEG artifacts such as eye blinks were visually identified and removed along with those components representing electrical stimulation artifacts [23]. Back-projected single trials were again screened for residual artifacts. On average, 4% of all trials were rejected. Data were then re-referenced to the average reference of all channels, the baseline (−200 to 0 ms) was subtracted, and a 30 Hz low-pass filter was applied.

### EEG data analyses

We used a global data-driven topographic neuroimaging approach [34] to examine the spatio-temporal brain mechanisms contributing to effects of high- *vs.* low-calorie food viewing on subsequent taste processing. All EEG results reported are relative to the physically identical taste stimulation preceded by food images.

#### ERP analyses

**Figure 2:**
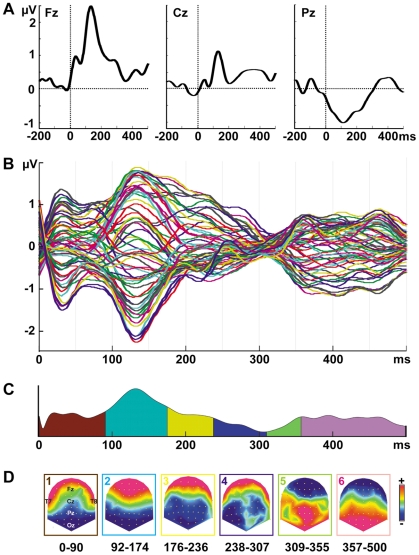
Event-related responses. (A) Exemplary group-averaged ERP waveforms from electrode Fz, Cz, and Pz. Taste onset is at time 0 ms. (B) Time course of group-averaged ERP waveforms from 64 electrodes to the taste stimuli (taste onset = at 0 ms) averaged across all conditions. (C) The temporal extent of the topographic map series is indicated as colored segments under the global field power curve. (D) Topographic maps (scaled to the maximum; top view) correspond to the temporal succession of segments in (C). Hot and cold colors reflect positive and negative ERP amplitudes, respectively.

In a first step, event-related potentials (ERPs) were computed for single electrodes and plotted, averaged across experimental conditions and participants, to visualize the ERP waveform data (Fig. 2A and B).

#### Topographic cluster analysis

Grand-averaged ERP waveforms from 0–500 ms after taste onset were clustered into periods of common topography to identify the predominant topographic maps and their temporal sequence using a topographic atomize and agglomerate hierarchical cluster (T-AAHC) analysis [35] as implemented in the Cartool software. Model parameters were set such that clusters spatially correlating above 95% were merged. We further applied the constraint that each topographic template map has to be observable for at least 20 ms in the group-averaged data. The optimal number of topographic template maps was determined using a modified Krzanowski-Lai criterion [36]. The cluster analysis provides a data-driven means to summarize ERP data by a limited number of topographic maps and their temporal occurrence.

#### Source estimation (LAURA)

The stable ERP periods as obtained from the topographic cluster analysis were used to define the time windows of interest for statistical comparisons in source space. Relevant statistical comparisons were computed between taste stimuli preceded by high-calorie vs. low-calorie cues. Neural sources of the taste EEG responses were estimated using the local autoregressive average (LAURA) distributed linear inverse solution [37]. The lead field was calculated on a spherical head model with anatomical constraints (SMAC model) [38]; it contained 3005 solution points equally distributed within the gray matter of the cerebral cortex and limbic structures of the Montreal Neurological Institute's (MNI) 152 average brain. The inverse solution was first estimated for each of the 3005 nodes for each time window of interest determined by the topographic cluster analysis, so that scalar values (indicating activation strength in µV/mm^3^) could be extracted from each node. Then, paired Student t-tests were calculated at each source space node using the variance across time points within the period of interest. In order to correct for multiple comparisons, effects were considered significant only when a minimum of 12 neighboring nodes was observed with p≤0.001 (t_1,13_≥5.5). The results of the source estimations were then averaged across time points of the respective period of interest and the average was rendered on the MNI brain with the Talairach and Tournoux coordinates of the maximal statistical differences indicated [39].

Pearson's correlation coefficients were used to test for associations between the changes in brain activation induced by high- *vs.* low-calorie food viewing on subsequent taste perception and changes in participant's taste ratings between these conditions. The analysis was spatially focused on the key brain areas that have been previously associated with taste valuation, namely the OFC [20] and the insula [22]. Activations within the insula/opercula and OFC/ACC were observed only during the 92–174 ms period (left and right insula), during the 176–236 ms period (OFC/ACC), and during the 357–500 ms period (right insula). The maximum neural source strength was thus extracted for each participant from these areas during the time windows identified. In other words, the source node exhibiting the maximum activity for taste preceded by high- and low-calorie image viewing within these regions and during these time periods was selected for each participant. Then, the difference in source activation between taste stimuli preceded by high- and low-calorie food cues was calculated. Finally, the correlation between the difference in pleasantness ratings and the difference in the source strength was computed for each of the four combinations of area and time periods of interest. The α level was a priori set to 0.05.

## Results

### Behavioral data

The mean taste detection threshold was 7.1 db (range: −6 to 12 db), which is within the normal range [40]. In line with our previous findings [23], [41], participants reported predominantly metallic (32%), sour (16%) and rusty (11%) tastes during the threshold assessment. Participants' ratings for taste pleasantness and intensity during the EEG sessions were compared using Wilcoxon signed-rank tests. Overall, subjects reported a neutral, slightly pleasant, experience in response to taste stimuli with mean pleasantness scores above 2.5 on the 5-point rating scales. Taste stimuli were perceived significantly more pleasant (Z = −2.43, p = 0.01; Wilcoxon singed-rank test, 2-tailed) when preceded by images of high-calorie food (mean = 2.9, SEM = 0.15) than images of low-calorie food (mean = 2.75, SEM = 0.14). Perceived taste intensity was marginally augmented when preceded by high-calorie *vs.* low-calorie food pictures but this augmentation failed to reach significance (Z = −1.73, p = 0.09; Wilcoxon singed-rank test, 2-tailed).

### ERP analyses

Figure 2A depicts the group-averaged ERP waveforms evoked by the taste at 64 electrodes. Six ERP map clusters accounting for 96.5% of the variance were identified (Fig. 2C). The temporal extent of each topographic map is indicated as colored segments under the global field power (GFP) curve. The topographic maps corresponding to each segment are displayed in Figure 2D. Map 1 was observed from 0–90 ms relative to taste onset and was characterized by a centro-posterior negativity and a fronto-lateral positivity. During the period of Map 2 (92–174 ms), a pronounced deflection with a maximum over frontal and a minimum over posterior electrodes constituted the P1 ERP component. The P1 was followed by a negative component, corresponding to the N1, with a minimum over posterior and central electrodes (Map 3, 176–236 ms). After two transition maps between 238–355 ms, a late positive complex (LPC) was observed with the maximum over posterior and parietal electrodes (Map 6, 357–500 ms).

### Effects of high-calorie versus low-calorie food cues on taste processing

**Figure 3:**
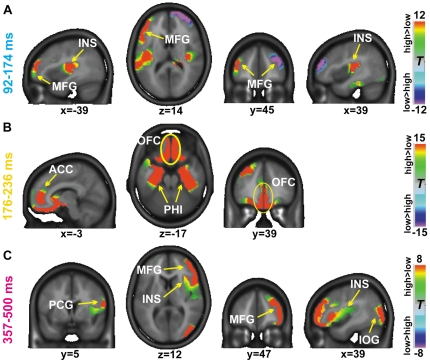
LAURA source estimations. Statistical contrasts of LAURA source estimations computed for taste stimuli when preceded by high-calorie versus low-calorie images. Time periods were obtained from the topographical cluster analysis. Color scales represent *t*-values with positive values indicating stronger activity during taste perception preceded by high-calorie than low-calorie images and negative values indicating increased activity when taste was preceded by low- as compared with high-calorie images. MFG = middle frontal gyrus, INS = insula, ACC = anterior cingulate cortex, OFC = orbitofrontal cortex, PHI = parahippocampal gyrus, PCG = postcentral gyrus, IOG = inferior occipital gyrus.

To identify brain areas that reveal differential activation as a function of the image category preceding the taste, we contrasted the neural source activity during taste perception following the viewing of high-calorie food as opposed to low-calorie food images. For this purpose, the obtained stable topographic cluster periods (cf. Figure 2) served as input for the neural source estimation algorithm (LAURA). Significant differences between taste perception following high-calorie and low-calorie food viewing were obtained over the three time intervals of interest, *i.e.* from 92–174 ms, from 176–236 ms and 357–500 ms. Figure 3 illustrates these differences with the Talairach coordinates of activation difference maxima indicated. Positive *t*-values in Figure 3 evince higher neural source activity when the taste stimuli were preceded by images depicting high-calorie food as compared to low-calorie food images and *vice versa*.

Over the time interval of Map 2 presence (92–174 ms; cf. Figure 2), we observed higher activity in the transition between the right insular gyrus and FOP transition (maximum x,y,z coordinates: 35, −22, 15; t = 13.92), as well as in the left FOP (−44, −16, 14; t = 13.88) when high-calorie as compared to low-calorie images preceded the taste. Furthermore, the left middle frontal gyrus (−47, 32, 19; t = 17.07) and the right parahippocampal gyrus (0, −34, −9; t = 14.04) yielded stronger activation to taste preceded by high-calorie viewing. In contrast, the right middle frontal gyrus (22, 63, 14; t = −18.91) responded stronger to taste stimuli preceded by low calorie images than high calorie images.

Over the consecutive time period of Map 3 presence (176–236 ms), prominent differential activity was observed in the medial orbitofrontal gyrus (−3, 39, −13; t = 19.75) and the adjacent ACC. Moreover, the left superior and middle frontal gyrus (−27, 49, 26; t = 19.65) exhibited stronger activation when following high-calorie as opposed to low-calorie food images preceded the taste stimulus. In both hemispheres, the parahippocampal and fusiform area (left: −26, −6, −25; t = 16.02; right: 26, −6, −25; t = 13.55) and adjacent uncus yielded significantly stronger activation when high- *vs.* low-calorie food was presented prior to the taste.

Between 357–500 ms (interval of Map 6 presence), major clusters of activation differences were observed within the right middle frontal gyrus (49, 45, 3; t = 12.35), the right insula (35, 9, 9; t = 7.40) and the parietal operculum and postcentral gyrus (63, −21, 30; t = 10.30), as well as in the right inferior occipital gyrus (43, −79, 12; t = 11.17). In all these regions, neural source strength was higher when high-calorie foods were viewed before taste stimulation as opposed to low-calorie foods.

**Figure 4:**
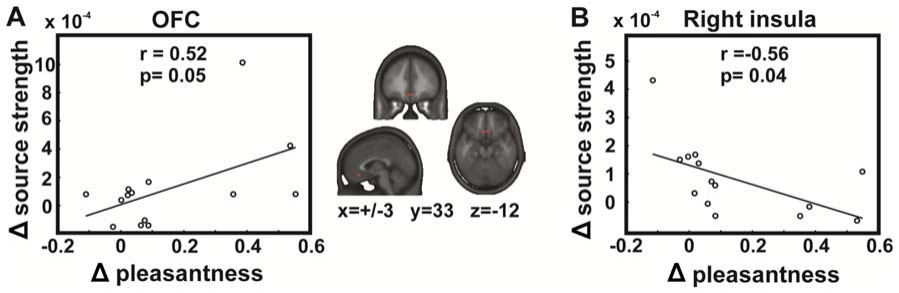
Correlations between changes in source strength and taste pleasantness ratings. Correlations were determined between changes in brain activation (*i.e.* high-calorie minus low-calorie) in the OFC and right anterior insula and changes in participant's taste ratings (*i.e.* high-calorie minus low-calorie). The source strength was determined at the voxel exhibiting the maximum activity within the region of interest for each participant during the 176–236 ms period for the OFC and during the 357–500 ms period for the right insula.

Associations between activation differences to taste preceded by high- *vs.* low-calorie food cues in regions of interest (insula/FOP and medial OFC) and respective alterations in taste pleasantness ratings were determined over the 92–174 ms (bilateral insula/FOP), the 176–236 ms (medial OFC), and the 357–500 ms (right insula/FOP) time intervals. Initial food cue-induced activation differences in the right and left insula (92–174 ms) were not significantly correlated with the differences in taste pleasantness. However, the activation differences observed in the medial OFC over the successive time period from 176–236 ms correlated positively with changes in taste pleasantness (Figure 4; r = 0.52, p = 0.05). The activation maxima converged across participants at the Talairach coordinates x = +/−3, y = 33, z = −12. Moreover, over the 357–500 ms interval, the source strength differences derived from individuals' activation maxima within the right insula/FOP correlated negatively with changes in taste pleasantness (r = −0.55, p = 0.04).

## Discussion

Our results show that the viewing of high-calorie foods increases the pleasantness and related cortical activation of subsequent hedonically neutral taste, relative to low-calorie food items. Most importantly this was observed with constant physical characteristics of taste stimulation throughout the experiment. The changes in neural activation encompassed the insula and FOP, ventral prefrontal cortex (including OFC), and ACC, *i.e.* regions previously associated with the hedonic evaluation of food. In particular, high-calorie cues induced an early modulation of the taste response in the insula/FOP within ∼100 ms, followed by alterations in the OFC at ∼180 ms, and in the insula/FOP around 360 ms, always relative to taste onset. Within 180 ms after taste onset, increases in pleasantness ratings of the taste preceded by high-calorie versus low-calorie cues were positively correlated with changes in activation strength in the OFC. Increased pleasantness was furthermore inversely related to activity in the right insula/FOP from 357–500 ms, possibly indicating a revaluation of the taste percept. These findings provide novel and temporally fine-grained information on the involvement of particularly the insula and OFC in the modulation of taste perception and evaluation by visual calorie cues.

Hemodynamic imaging studies have linked insular activation predominantly with subjective taste intensity while modulations in the OFC have been mostly related to taste pleasantness [13], [42]. Moreover, evidence suggests that both the insula and OFC serve as convergence zones for the representation and integration of information across senses. For example, the insula has been associated with bodily interoception based on multimodal cues [43]–[45]. On the other hand, the OFC has been directly related to the integration of visual and gustatory information [46] as well as food valuation and selection [47], [48].

Our current results show a dynamic and fast interplay of the insula, orbitofrontal, and anterior cingulate regions pointing toward an interactive integration of visual energy cues and taste. Given the established anatomical connectivity between the insula, ACC, and frontal and superior temporal cortex in primates [49], [50] and between the insula and ACC in humans [51] such finding seems reasonable. Despite the need to further elaborate on the anatomical connections, functional studies agree that the insula, OFC, and ACC are involved in reward-related processing including food and taste.

The differences in initial insular activation (92–174 ms) observed here were not correlated with changes in taste pleasantness and thus possibly reflect the formation of a precursor for valuation rather than valuation itself. In line with our hypothesis, research has demonstrated that taste representations can already be modulated by contextual cues during early levels of encoding [21]. Unexpectedly, the early effect in our study occurred without concomitant changes in taste intensity although previous studies have linked modulations of insular activity with perceived taste intensity [13], [52]. Most likely, differences between studies are due to the threshold approach taken to detect activations in statistical parametric maps. However, it has also been suggested that separate representations of the sensory and hedonic aspects of taste are hosted in the insula [46], [53]. As highlighted, the taste in our study was physically constant and the early insular activation differences changed as a function of the calorie content of the visual food cue without an association with changes in perceived taste intensity and/or taste pleasantness. Thus, this effect might also be related to expectations about the imminent taste, elicited by the visual cue. This alternative explanation of our findings is corroborated by a recent observation from functional neuroimaging that expectations about taste modulate activity in the insula [54]. We further propose that the differential activations in the left middle frontal cortex over the same time interval designate integration [55] of the visual energy cue and the taste stimulus, mediated by anatomical connections between the frontal and the anterior cingulate cortex.

Over the time interval from 176–236 ms, greatest activation differences for taste sensations altered by visual food cues were found in the OFC and adjacent ACC. These areas are involved in the hedonic evaluation of taste [16], [18], [56]. In addition, Grabenhorst and co-workers (2008) reported activation in the OFC but not in the insula, when taste pleasantness increased after the presentation of a positive word label [20]. In line with this, the taste activation differences in our study were associated with augmentations in taste pleasantness between taste stimulation preceded by high- *vs.* low-calorie food cues, and can be interpreted as the first level of processing that directly relates to the augmented hedonic experience.

Previously, it has been shown that the insula and adjacent opercula discriminate not only taste qualities but also tastes of different hedonic value [54], [56], [57]. Our findings especially evince that changes in pleasantness for tastes preceded by high- *vs.* low-calorie food cues co-vary with smaller changes in right insula/FOP activation (357–500 ms). This is consistent with the data by Nitschke and co-workers (2006) showing greater activations in the right insula when an aversive taste was perceived as more unpleasant than it actually was due to modulations of expectancy by non-verbal visual cues. In other words, the authors found that the right insula responds to aversive and unpleasant tastes, and that an increase in pleasantness, *i.e.* a decrease in unpleasantness in Nitschke's study (2006), of physically identical tastes induced by visual context cues leads to decreased activation in this region. In contrast, Nitschke could not show that pleasant tastes that were perceived as more pleasant following a visual cue yielded changes in insular activations. The authors attributed the lack of result to their study design, *i.e.* the randomized presentation of pleasant and unpleasant tastes. In our study, increases in pleasantness of a neutral, slightly positive, taste following high-calorie visual cues coincide with dampened activation in the right insula that, in turn, reduces the source strength differences to tastes preceded by high-calorie as opposed to low-calorie cues. We speculate that this effect reflects a revaluation of interoceptive taste awareness based on changes in the hedonic appraisal of the visual context cue or the diminution of aversive feelings, which is supported by findings showing that the anterior insula is implicated in coding disgust and in the experience of emotion as it is part of an emotional ‘salience’ network [45], [58].

Further prominent and sustained activations were observed in the parahippocampal gyrus (PHI), which has recently been proposed to mediate visual-contextual associations [59], [60]. Together with the observed increases in activation in the visual cortex for combinations of high-calorie cues and taste our data possibly indicate that the PHI also mediates visual-gustatory associations. However, we cannot exclude that PHI activations are due to the behavioral food – non-food discrimination task that participants had to perform after taste stimulation.

Why did we use electric taste instead of common taste qualities, *e.g.* a sweet solution? Electric taste is perceived as neutral in terms of its pleasantness when presented at the level of individual detection thresholds and it induces brain activations in gustatory areas (bilateral anterior insula, medial orbitofrontal cortex) [23]. While electric taste stimulation may limit the generalization of our findings to other tastes qualities, it yet provides a unique means to assess effects of visual food cues without confounds due to learned food-taste associations as they exist for basic tastes. Future studies should develop designs suitable to assess the relevance of our findings in settings in which participants interact, for example, with visual cues (e.g., advertisements) during the consumption of ecologically relevant taste stimuli or even complex foods. Such a design is hampered mainly by technical difficulties in stimulus control and the relatively high number of stimulus repetitions required for ERP analyses.

In conclusion, the present results provide evidence that high-calorie food cues enhance the hedonic evaluation of subsequently presented tastes. We suggest that the early calorie-dependent alterations in taste perception encompassing primary taste areas point to their role in taste evaluation in the presence of and/or as a consequence of visual representations of high-calorie foods. Later activation differences in the OFC and in the right insula were found to mediate subjective pleasantness likely through an enhancement of taste hedonics and revaluation of interoceptive taste awareness, respectively. The present study thus provides novel insights into cross-modal sensory interactions underlying taste and, in extension, probably also food evaluation and consumption. Future studies will have to elucidate to what extent the brain regions shown to be involved in visual-gustatory interactions could account for regulation of appetite and food intake control in real world settings.
